# Familial hypobetalipoproteinemia in pediatric patients with fatty liver: an under-recognized cause

**DOI:** 10.3389/fmed.2026.1720731

**Published:** 2026-04-22

**Authors:** Nurit Loberman Nachum, Eyal Shteyer, Hofit Cohen, Odelia Chorin, Maya Granot, Batia Weiss

**Affiliations:** 1 Pediatric Gastroenterology and Nutrition Unit, Edmond and Lily Safra Children’s Hospital, Sheba Medical Center, Ramat Gan, Israel; 2 The Gray Faculty of Medical and Health Sciences, Tel Aviv University, Tel Aviv, Israel; 3 Juliet Keidan Institute of Pediatric Gastroenterology and Nutrition, Shaare Zedek Medical Center, Jerusalem, Israel; 4 The Faculty of Medicine, The Hebrew University of Jerusalem, Jerusalem, Israel; 5 Strassburger Lipid Center, Sheba Medical Center, Ramat Gan, Israel; 6 The Danek Gertner Institute of Human Genetics, Sheba Medical Center, Ramat Gan, Israel

**Keywords:** apolipoprotein B, fatty liver, genetic, low-density lipoprotein, microsomal triglyceride transfer protein, pediatric

## Abstract

**Introduction:**

Fatty liver is a leading cause of chronic liver disease in children, most often due to metabolic dysfunction-associated steatotic liver disease (MASLD). Fatty liver in lean or younger children may indicate underlying metabolic or genetic disorders, such as familial hypobetalipoproteinemia (FHBL). We examined the importance of considering familial hypobetalipoproteinemia (FHBL) in children presenting with fatty liver.

**Methods:**

This case series includes seven patients with FHBL from three families, treated at two medical centers. Clinical, laboratory, imaging, and genetic data were collected from the medical records.

**Results:**

Four patients were male. The mean age at diagnosis was 15.3 years (range: 4–38). All the index patients were children or adolescents presenting with fatty liver. Genetic evaluation revealed biallelic variants in the microsomal triglyceride transfer protein (*MTTP*) gene in two siblings and heterozygous variants in the apolipoprotein B (*APOB*) gene in five patients. The mean body mass index was 27 kg/m^2^, and two patients had a BMI above 30 kg/m^2^. The median follow-up (FU) time was 12 months (interquartile range [IQR]: 1–57 months). Liver enzymes were elevated in three patients (range: 50–300 IU/L); median aspartate aminotransferase and alanine aminotransferase levels were 30 IU/L (IQR: 26–40.75) and 32 IU/L (IQR: 24.5–74), respectively. The median triglyceride, low-density lipoprotein, and apolipoprotein B levels were 74 mg/dL (IQR: 62–125), 55.3 mg/dL (IQR: 30–84.5), and 39.5 mg/dL (IQR: 35.2–43), respectively.

**Conclusion:**

FHBL should be considered a potential diagnosis in children with fatty liver and may coexist with other contributing etiologies such as obesity.

## Introduction

Fatty liver is increasingly recognized as a significant cause of chronic liver disease in pediatric populations and is commonly associated with metabolic dysfunction-associated steatotic liver disease (MASLD). MASLD affects up to 15–20% of children and an even higher proportion of children with obesity (1). Fatty liver disease has been reported as the most rapidly increasing indication for liver transplantation among young adults (2, 3). MASLD results from the pathologic accumulation of fat in hepatocytes (4). However, in younger children, particularly those who are lean, alternative diagnoses, including metabolic and genetic disorders, should be considered. These patients are usually diagnosed incidentally due to elevated liver enzymes on routine blood tests, followed by imaging that demonstrates variable degrees of fatty liver. An important genetic etiology in this context is familial hypobetalipoproteinemia (FHBL) (5).

Familial hypobetalipoproteinemia is an autosomal codominant disorder characterized by low plasma levels of low-density lipoprotein (LDL), typically below the fifth percentile (58 mg/dL) (6), and low plasma apolipoprotein B (ApoB) levels below the fifth percentile. Low plasma ApoB levels have been reported as <55 mg/dL in adult men and <45 mg/dL in adult women (7, 8). The prevalence of the heterozygous form of FHBL was estimated as 1:1000–1:3000 (9). Over 60 pathogenic variants in the *ApoB* gene have been reported as causative of FHBL (8), mostly splicing or nonsense mutations with a loss-of-function effect (7)^1^. Although patients with FHBL may have a reduced risk of cardiovascular diseases (10), they have increased risk for liver steatosis, fibrosis, and cancer (11, 12). Early diagnosis of FHBL can facilitate the implementation of appropriate measures to prevent advanced liver disease (12).

ApoB is a large structural glycoprotein required for the assembly and secretion of lipoproteins, with two main isoforms: ApoB-100 and ApoB-48. ApoB-100 is produced in the liver and plays a central role in the formation and secretion of very low-density lipoprotein, intermediate-density lipoprotein, LDL, and lipoprotein (a). ApoB-100 also functions as the ligand for the LDL receptor (13, 14). ApoB48 is a truncated form of ApoB-100 that comprises 48% of the 100 amino acid sequence. It is expressed in the intestine and is secreted by enterocytes as a component of chylomicrons and their remnants (10, 13, 14). The assembly and secretion of ApoB-containing lipoproteins depend on the microsomal triglyceride transfer protein (MTTP), a heterodimeric protein located in the endoplasmic reticulum. MTTP facilitates the transfer of triglycerides and cholesterol esters to ApoB during lipoprotein assembly in both hepatocytes and enterocytes (15–17). Biallelic pathogenic variants in the *MTTP* gene encoding the MTTP protein lead to abetalipoproteinemia (ABL; OMIM 200100). This rare autosomal recessive disorder is characterized by the intracellular degradation of ApoB, the absence of ApoB-containing lipoproteins, and severe fat-soluble vitamin malabsorption. The flip side of this process is lipid accumulation in hepatocytes and in intestinal epithelial cells, along with marked hypolipoproteinemia (17–19).

Familial hypobetalipoproteinemia is part of the ABL spectrum of disorders caused by variants that retain residual ApoB function. The incidence of progression from hepatic steatosis to clinically significant cirrhosis is unknown (9).

The prevalence of obesity and metabolic dysfunction-associated fatty liver disease has been increasing consistently in the pediatric population and may coexist with FHBL. Consequently, FHBL may be overlooked in individuals diagnosed with obesity and metabolic syndrome. We suspect that FHBL is underdiagnosed in the pediatric population, particularly as hepatocellular enzyme testing is rarely performed in children of normal weight. Moreover, individuals with obesity may have co-existing FHBL, which is overlooked due to the focus on obesity and metabolic syndrome. Thus, the true prevalence of FHBL is likely higher than currently reported. In this study, we present three families with diagnoses of FHBL and aim to raise awareness and maintain clinical suspicion of this disease among pediatric patients.

## Materials and methods

### Patients and data

This case series includes seven patients from three unrelated families who presented at two medical centers between 2018 and 2024 (Table 1). From the medical records of each patient, the data retrieved included demographic and clinical characteristics and laboratory, imaging, and genetic testing results. Demographic and clinical characteristics included age, gender, body mass index (BMI), clinical symptoms, the reason for the investigation, and family history. Laboratory parameters included liver enzymes, bilirubin, serum albumin, chemistry, lipid profile, ApoB plasma levels, and fat-soluble vitamin levels. The radiologic assessments retrieved included abdominal ultrasound (US), liver elastography, FibroScan, and magnetic resonance cholangiopancreatography (MRCP). A liver biopsy was undertaken after laboratory and imaging studies were inconclusive regarding the etiology of hepatic steatosis and prior to identification of the diagnosis by genetic testing.

**Table 1:** Demographic and clinical characteristics of the 7 patients with familial hypobetalipoproteinemia included in the case series.

	Demographic and clinical characteristics									
Patient #	Family#	Medical center	Sex	Age at first FU (years)	FU (y)	BMI (kg/m2)	BMI (*z*-score)	Regular medications at diagnosis	Reason for investigating	Family history of fatty liver
1	1	Sheba	M	17	3.5	24.45	+0.9	NO	Elevated liver enzymes	Father, brother
2		Sheba	M	17	<1	38.27	+2.6	NO	Family history	Father, brother
3	2	Sheba	M	14.9	6.5	21.35	+0.5	Infliximab	Incidental finding in MRI D/T Crohn’s Disease	Mother, sister
4	3	Shaare Zedek	F	38	1	18.59		NO	Family history	Offspring
5		Shaare Zedek	M	12	6	33.03	+2.4	Risperidone	Elevated liver enzymes	Mother, sisters
6		Shaare Zedek	F	4	<1	NA	NA	NO	Family history	Mother, brother
7		Shaare Zedek	F	4	<1	NA	NA	NO	Family history	Mother, brother

FU, follow-up, BMI, body mass index, NA, not available, MRI, magnetic resonance imaging.

For some parameters, the follow-up period was limited, and data points are missing (Supplementary Table 1). This is because certain patients were diagnosed after a family member was identified and attended only one appointment with the physician to complete the genetic testing and to obtain their diagnosis. As these patients were asymptomatic, they did not complete some blood tests, imaging, or biopsy examinations, nor did they return for follow-up appointments. Missing data were addressed as not available.

### Genetics

Genetic analysis was performed using exome sequencing at the Bioinformatics Unit of the Sheba Medical Center (*n* = 3) and using panel testing at Pronto Laboratories (*n* = 1) using standard protocols (Table 2). Variant filtering, annotation, and classification were conducted according to the guidelines of the American College of Medical Genetics and Genomics (20). Segregation analysis was performed within the family following the identification of causal variants by Sanger sequencing.

**Table 2:** Genetic characteristics and identified mutations in the seven patients included in the case series.

Genetics and patient mutations							
Patient	Gene	Genomic position	Patient’s zygosity	Type mutation	Variant frequency (GnomAD)		ACMG
1	*MTTP*	Chr4: 99,622,756	Maternal heterozygote	G> > T	0.24%	NM_000253.4 c.2593G > T p.Gly865Ter STOPGAIN	Pathogenic
	*MTTP*	Chr4: 99,606,880	Paternal heterozygote	G> > T	0.0125%	NM_000253.4 c.1477G > T p.Ala493Ser Missense	VUS
2	*MTTP*	Chr4: 99,622,756	Maternal heterozygote	G> > T	0.24%	NM_000253.4 c.2593G > T p.Gly865Ter STOPGAIN	Pathogenic
	*MTTP*	Chr4: 99,606,880	Paternal heterozygote	G> > T	0.0125%	NM_000253.4 c.1477G > T p.Ala493Ser Missense	VUS
3	*APOB*	Chr 2: 21,005,468	heterozygote	A> > C	0.0016%	NM_000384.3 c.11400 T > G p.Tyr3800Ter STOPGAIN	Likely pathogenic
4	*APOB*	Chr2: 21,006,154	Heterozygote	G> > A	0	NM_000384.2 c.10714C > T; p.Gln3572 Ter	Pathogenic
5	*APOB*	Chr2: 21,006,154	Heterozygote	G> > A	0	NM_000384.2 c.10714 > T; p.Gln3572	Pathogenic
6	*APOB*	Chr2: 21,006,154	Heterozygote	G> > A	0	NM_000384.2 c.10714 > T; p.Gln3572	Pathogenic
7	*APOB*	Chr2: 21,006,154	Heterozygote	G> > A	0	NM_000384.2 c.10714 > T; p.Gln3572	Pathogenic

MTTP, microsomal triglyceride transfer protein; APOB, apolipoprotein B; ACMG, American College of Medical Genetics and Genomics; VUS, variant of uncertain significance.

### Statistical analysis

Non-parametric data are presented as means with standard deviations or medians and interquartile ranges (IQR).

### Ethics statement

The study was approved by the Ethics Committee of all the institutions (No. 8471-21-SMC).

## Results

The index patients from each of the three families were referred due to the incidental findings of fatty liver in the absence of obesity, hyperlipidemia, or metabolic diseases. Their evaluations for other causes of fatty liver, including hepatitis C, Wilson’s disease, autoimmune hepatitis, and celiac disease, yielded normal results. This prompted evaluations for FHBL. The other four patients were diagnosed through family screening following the index patients.

Four patients were male. The mean age at diagnosis was 15.3 (range: 4–38) years. The median follow-up time was 12 months (IQR: 1–57). While three patients had a BMI within the normal range, two patients had an elevated BMI (z-scores: 2.6 and 2.4) (Table 1). Liver enzymes were elevated in only three patients. ALT levels were 50 IU/L, 98 IU/L, and 300 IU/L for patients one, two, and five, respectively (Table 1). The median AST and ALT levels were 30 IU/L (IQR: 26–40) and 32 IU/L (IQR: 24.5–74), respectively (normal range: 0–45 IU/L). The mean serum albumin level was 4.74 g/dL ± 0.17. Prothrombin time and the international normalized ratio were within normal limits in all patients. The median (IQR) triglyceride, LDL, and ApoB levels were 74 mg/dL (62–125), 55 mg/dL (30–84.5), and 40 mg/dL (35–43), respectively (normal ranges: 30–150 mg/dL, below 130 mg/dL, and 40–125 mg/dL, respectively). Fat-soluble vitamin levels were normal for all four patients for whom these were examined.

Abdominal US showed mild to moderate steatosis in all the patients. Elastography was performed in two patients and was F1 (*n* = 1) and F0 (*n* = 1). FibroScan was conducted in two other patients and was F0 for both. In one patient, liver MRI/MRCP testing revealed a fatty liver with focal fatty sparing and normal bile ducts, and in another patient with Crohn’s disease, the MRCP showed a normal liver with focal stenosis in the bile duct.

As part of investigating abnormal liver enzymes and hepatic steatosis, while the primary genetic diagnosis was unknown, a percutaneous US-guided liver biopsy was performed in two patients. Patient one exhibited severe steatosis with minimal inflammation, sinusoidal and periportal fibrosis, and cholangiolar proliferation. Patient five showed moderately active steatohepatitis, with portal and sinusoidal fibrosis and severe macrovesicular steatosis.

Genetic testing revealed biallelic variants within the *MTTP* gene in two siblings: c.2593G > T, p. (Gly865Ter), classified as pathogenic, and c.1477G > T, p. (Ala493Ser), classified as a variant of unknown significance (Table 2). Parental testing confirmed trans-configuration. Five patients carried heterozygous pathogenic *APOB* variants: c.10714C > T; p. (Gln3572Ter) recurred in four individuals from the same family and c.11400 T > G p. (Tyr3800Ter) recurred in one individual.

Three patients were followed for more than 3 years, during which no signs of liver function decline or abnormalities in liver imaging were observed.

## Discussion

In this case series of seven patients with genetically confirmed FHBL, the three index patients were identified due to elevated liver enzymes or fatty liver detected during routine testing, whereas the remaining four patients were diagnosed through family cascade screening.

Pathogenic variants in the *ApoB* and *MTTP* genes can cause either abetalipoproteinemia or hypobetalipoproteinemia (21). Carriers of heterozygous pathogenic variants typically present with hypobetalipoproteinemia, often accompanied by mild elevation of liver enzymes and hepatic steatosis. In contrast, patients with biallelic variants develop abetalipoproteinemia, characterized by fat intolerance, steatorrhea, diarrhea, fat malabsorption, lipid accumulation in enterocytes, failure to thrive, and deficiencies in fat-soluble vitamins, and neurologic and ocular manifestations. Additional genes implicated in FHBL include *PCSK9*, *SAR1B,* and *SARA2* (8). Zhang et al. reported that the analysis of common single-nucleotide polymorphisms may help predict lipid levels in the general population, and genetic prediction of cholesterol levels may reduce the risk of cirrhosis (22).

Three of our patients had LDL levels below the fifth percentile (58 mg/dL). Five patients exhibited low levels of ApoB. Two patients presented with elevated triglyceride levels, while two others displayed low high-density lipoprotein levels. Moreover, two patients were overweight, with a BMI exceeding 30 kg/m^2^ (*z*-scores: 2.4 and 2.6); of these two patients, one had hypertriglyceridemia. This patient, for example, could have been diagnosed with metabolic syndrome as the cause of fatty liver, potentially overlooking other etiologies such as FHBL. No patient in our study had diabetes; however, other studies have shown that insulin resistance independently promotes hepatic steatosis and increases hepatic production of ApoB-containing lipoproteins, thereby attenuating the expected lipid profile in hypobetalipoproteinemia (23). Notably, individuals with FHBL may exhibit disproportionately increased liver fat for a given BMI compared to controls (24), suggesting that FHBL amplifies obesity-associated steatosis and, without genetic testing, may be clinically recognized as MASLD. Moreover, obesity-related ApoB–LDL cholesterol discordance may further obscure the diagnostic signal of FHBL, leading to under-recognition unless genetic testing is performed.

Although individuals with FHBL are predisposed to fat-soluble vitamin malabsorption (25), none of the patients in our series demonstrated deficiencies in fat-soluble vitamins or related clinical symptoms.

Prior to genetic confirmation, liver biopsies were performed in two patients. In patient one, histology demonstrated severe macrovesicular steatosis with minimal inflammation, sinusoidal and periportal fibrosis, and cholangiolar proliferation, despite only mildly elevated liver enzyme levels. In contrast, patient five, who exhibited more pronounced transaminase elevation, showed moderately active steatohepatitis with portal and sinusoidal fibrosis and severe macrovesicular steatosis. These histologic findings are consistent with previously reported features of FHBL (10, 24, 26) and illustrate that biochemical abnormalities do not always correlate with the severity of histologic liver involvement. During follow-up, patient one showed improvement with normalization of liver enzymes; however, abdominal ultrasonography remained unchanged, demonstrating persistent hepatomegaly with mild to moderate steatosis.

Two of our patients harbored the following variant in the *MTTP* gene: c.2593G > T, p. (Gly865Ter). This variant is present in 0.25% of the Jewish Ashkenazi population and was reported as a founder Ashkenazi variant (26). The substitution in the variant creates an early termination codon, whereas the truncated protein is expected to undergo nonsense-mediated decay. The variant was reported multiple times and classified as pathogenic in ClinVar (RCV000015311). In a homozygous state, the manifestations include a severe phenotype, encompassing pigmentary retinal degeneration, celiac enteropathy, ataxia, and neuropathy, with acanthocytosis in blood smear (26). Both carriers in our series harbored a missense variant in trans (compound heterozygotes): c.1477G > T p. (Ala493Ser). This variant is rare, with a maximal carrier frequency of 0.0125% in GnomAD, and there are no homozygotes. While originally classified as a variant of uncertain significance (VUS), configuration, familial segregation, and phenotypic data enabled us reclassify this variant as likely pathogenic (PM2, PP1, PM3, PP4). We hypothesize that residual functional protein activity resulting from the above alteration may partially explain the milder phenotypic presentation of the two aforementioned patients compared with patients harboring the p. (Gly865Ter) variant in a homozygous state. However, additional unknown genetic and non-genetic mechanisms may be involved.

Five of our patients presented with pathogenic *APOB* variants. The heterozygous variant c.10714C > T; p. (Gln3572 Ter) recurred in four family members. This variant is not present in population databases (gnomAD = 0) and creates a stop codon, with early termination of translation of RNA to protein. The truncated protein (exon 26/29) is expected to undergo nonsense-mediated decay. This variant is novel and has not been previously reported. One additional patient presented with the following heterozygous truncating *APOB* variant: c.11400 T > G, p. (Tyr3800Ter). This variant was reported to appear at a maximal frequency of 0.0016% in GnomAD (https://gnomad.broadinstitute.org) and does not have homozygotes. ClinVar categorized the variant as pathogenic (RCV003780969) and indicated the variant to be associated with low LDL levels (27). Based on the above findings, both variants were classified as pathogenic.

Our study underscores the importance of maintaining a high index of suspicion for FHBL in individuals presenting with hepatic steatosis who do not exhibit obesity or dyslipidemia.

To the best of our knowledge, no studies have compared patients with FHBL to a MASLD control group with respect to biochemical parameters, imaging findings, or histologic features. Accordingly, the aim of the present study is to raise clinical suspicion for FHBL when the overall picture is incongruent, particularly in both lean and obese patients whose laboratory evaluation reveals unexpectedly low lipid or apolipoprotein B levels. Genetic testing is a key facilitator in this process and may also help to differentiate patients who harbor other genetic predispositions. For example, recently, a common polymorphism in *PNPLA3*:c.444C > G, p. (Ile148Met) has been implicated as the strongest predictor for hepatic fat accumulation, steatohepatitis, fibrosis, cirrhosis, and hepatocellular carcinoma, with additional variants (for example, within the *TM6SF2, MBOAT7,* and *GCKR* genes) presenting a milder contributory effect that is independent of environmental factors (40802944). Hence, patient identification and genetic testing are prudent for risk stratification, prognostication, and treatment decision-making, as well as paving the pathway for novel drug developments. Furthermore, we have illustrated the effectiveness of genetic molecular testing, coupled with cascade testing of additional family members. This approach facilitates the provision of tailored precision medicine to all at-risk family members. While not demonstrated in our study, individuals with FHBL are known to be at increased risk for fat-soluble vitamin deficiencies and related symptoms, including ataxia, dysmetria, ophthalmological abnormalities, and bone loss (8). The absence of these symptoms in our cohort may be partially attributed to genotype–phenotype correlations or to the limited number of patients studied; however, prolonged follow-up is necessary to ascertain the associated risks.

Following the diagnosis of FHBL, regular long-term follow-up is recommended, including annual assessment of growth parameters (weight and height), developmental milestones, and neurological and gastroenterological status. To detect complications early, baseline and annual laboratory and imaging evaluations should include a lipid profile with apolipoprotein B and apolipoprotein A1, albumin, liver function tests, vitamin A, E, and D levels, international normalized ratio, complete blood count, vitamin B12, folate, calcium, phosphorus, uric acid, and an annual abdominal ultrasonography (28).

In conclusion, the diagnosis of FHBL should be considered among children and adults with hepatic steatosis and can coexist with other risk factors such as obesity. Awareness and screening for FHBL are imperative, given its unknown prevalence in individuals with MASLD. Monitoring for the development of cirrhosis and its complications in this population is necessary, and treatment options should be explored. This case series highlights the role of genetic evaluation of patients with fatty liver and its usefulness in identifying at-risk family members.

## Data Availability

The original contributions presented in the study are included in the article/Supplementary material, further inquiries can be directed to the corresponding author/s.

## Footnotes

1
https://www.ncbi.nlm.nih.gov/clinvar
