# Supplementary material for: Familial hypobetalipoproteinemia in pediatric patients with fatty liver: an under-recognized cause
Source: Front Med (Lausanne). 2026 Apr 22;13:1720731. doi: 10.3389/fmed.2026.1720731 (PMC13143782; doi:10.3389/fmed.2026.1720731)
Supplement: Supplementary file 1 [file Table_1.DOCX]

|  | | | | | | | |
| --- | --- | --- | --- | --- | --- | --- | --- |
| Available Data | | | | | | | |
| DATA  )normal range( | Patient 1 | Patient 2 | Patient 3 | Patient 4 | Patient 5 | Patient 6 | Patient 7 |
| Age (Y) | 17 | 17 | 14.9 | 38 | 12 | 4 | 4 |
| Weight (Kg) | 71.5 | 124 | 53.3 | 50 | 100 | 22 | 18 |
| Hight (m) | 1.71 | 1.8 | 1.58 | 1.64 | 1,74 | X | X |
| AST (0-45 IU/L) | 26 | X | 15 | 26 | 150 | 34 | 43 |
| ALT  (0-45 IU/L) | 50 | 98 | 23 | 32 | 300 | 22 | 26 |
| Albumin (3.5-5.5 g/dL) | 4.7 | 4.5 | 4.7 | 4.92 | 4.9 | X | X |
| INR | 1.01 | X | 1.19 | 0.96 | 1.02 | X | 1.07 |
| LDL (<130mg/dL) | 102.5 | 92 | 24 | 62 | 48.6 | X | 20.4 |
| ApoB  (40-125 mg/dL) | 71 | X | 20 | 44 | 39 | 34 | 40 |
| Vitamin A  (20-60 µg/dL) | 46 | X | 55.2 | 45 | 60.2 | X | X |
| Vitamin E μg/ml  (5-20 μg/mL) | 11 | X | 7.6 | 8 | 8.6 | X | X |
| Liver US | ⩗ | ⩗ | ⩗ | ⩗ | ⩗ | ⩗ | ⩗ |
| Liver MRI | ⩗ | X | ⩗ | X | X | X | X |
| Elastography | F1 | X | F0 | X | X | X | X |
| Fibroscan | X | X | X | F0 | FO | X | X |
| Liver biopsy | ⩗ | X | X | X | ⩗ | X | X |
| Genetic test | ⩗ | ⩗ | ⩗ | ⩗ | ⩗ | ⩗ | ⩗ |
| ALT, Alanine aminotransferase, AST, Aspartate transaminase, INR, International normalized ratio, LDL, Low density lipoprotein, ApoB, Apolipoprotein B, US, Ultrasonography, MRI, Magnetic resonance imaging. ⩗- Data is available, X- Data is not available. | | | | | | | |

**Supplementary Table 1 -** Availability of data for each patient
